# Quantitative Evaluation of Signal Intensity of Magnetic Resonance Images in Optic Neuritis

**DOI:** 10.2174/1874364100701010001

**Published:** 2007-07-06

**Authors:** Tadao Hanawa, Atsushi Mizota

**Affiliations:** 1 Department of Ophthalmology, Chiba Aoba Municipal Hospital, 1273-2 Aoba-cho, Chuoku, Chiba 260-0852, Japan; 2 Department of Ophthalmology, Juntendo University Urayasu Hospital, 2-1-1 Tomioka, Urayasu, 279-0021, Japan

## Abstract

We have evaluated the signal intensity of magnetic resonance (MR) images of the optic nerve quantitatively in 25 patients with unilateral acute optic neuritis (ON). MR imaging was performed with a 1.5 Tesla unit before treatment within 2 weeks after the onset of ON. Four coronal fat-suppressed T2-weighted images were obtained at 5, 10, 15, and 20 mm behind the eye. The ratio of the signal intensity of the MR images from the optic nerve to that of the white matter of the frontal lobe was calculated and we compared the signal intensity ratio of the affected eyes to the fellow healthy eyes. For statistical analysis paired t-test was used. At all 4 sections, the mean signal intensity ratio of the affected eyes is statistically significant higher than fellow eyes. The 11 patients showed optic disc swelling in the affected eyes and in all these 11 eyes had a higher signal intensity at 5 mm behind the eye compared with the fellow eye. From our present results, we cannot refer to the sensitivity of our method, because we did not use our present methods to other diseases. But with this method we think inter-image and inter-observer variability must reduce. Further studies are required about the sensitivity and the relation between the pathological condition of optic nerve and the signal intensity ratio.

## INTRODUCTION

The usefulness of magnetic resonance (MR) imaging in the diagnosis of optic neuritis (ON) was described as early as 1987 by Johnson *et al*. [1] using short time inversion recovery (STIR) sequences. With the technical advancement of MRI, there have been a number of studies that have not only demonstrated the usefulness of MRI but also shown an improvement in the certainty of the diagnosis [2-9].

Generally, MR imagings of the optic nerve have been interpreted by experienced neuroradiologists qualitatively. Thus in 1998, Jackson and his co-workers [7] studied the signal intensities in comparison to that of white matter and a combination of increased signal intensity and swelling in patients with ON and classified findings of optic nerve into 4 grades, grade 0 to 3. This normalization was used to remove the effects of inter-image and inter-observer variability. The purpose of their study was to examine the benefits of combined fat- and water-suppressed T2-weighted MRI for the diagnosis of optic neuritis.

Using their recommendations, we have investigated quantitatively the signal intensity of the MR images of the optic nerve at several points along the optic nerve in eyes with optic neuritis to determine the effectiveness of MRI in diagnosing and assessing optic neuritis.

## SUBJECTS AND METHODS

MRI was performed on 25 patients with unilateral acute ON before any treatment. There were 4 men and 21 women with a mean age of 36.4 ± 13.4 years. All patients did not have any history of ON in either eyes before. The diagnosis of ON was made on the basis of visual loss, papillary light reflex visual field defect, optic disc findings, and abnormal pattern visual evoked potentials (PVEP) in the affected eyes [10]. In the fellow eyes all these findings were normal. This research followed the tenets of the Declaration of Helsinki and informed consent was obtained from all patients after explanation of the nature and possible consequences of the study.

**Fig. (1):**
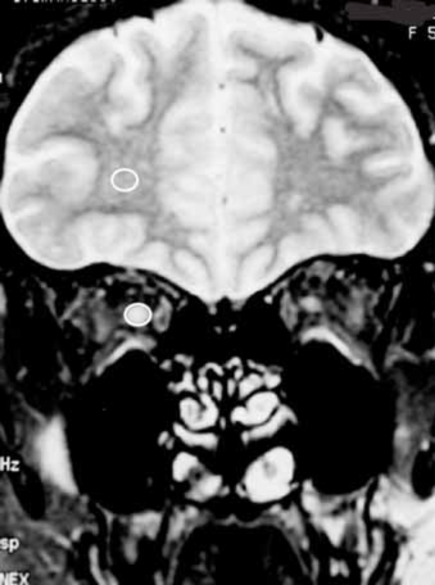
Signal intensity within white circle was measured in the one coronal MR image.

MR imaging was performed on a 1.5 Tesla unit (Signa Advantage, GE, Piscataway, NJ, USA) within 2 weeks after onset of ON. Coronal fat-suppressed T2-weighted images were obtained with 4000/100 (TR msec/TE msec) at 4 different plane, viz., 5, 10, 15, and 20 mm behind the eye. In all patients, there were no abnormalities in the frontal lobe. The signal intensity was measured in a 12 to 18 mm area of each slice of the optic nerve and white matter of the frontal lobe with Advantage Windows (GE, GE, Piscataway, NJ, USA) (Fig. 1). The size and shape of the measured area was adjusted to the size of optic nerve in each patient. To compare the signal intensities of the optic nerve, the ratio of signal intensity of optic nerve to that of white matter of the frontal lobe was calculated.

In the present study, we compared the signal intensity ratio of the affected eyes to the fellow healthy eyes. For statistical analysis paired t-test was used.

## RESULTS

**Fig. (2):**
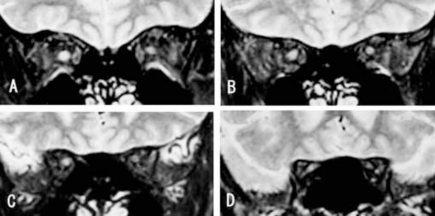
Typical MR image of a case with right ON. A: 5mm, B: 10mm, C: 15mm and D: 20mm behind eye.

The MR images of a 22-year-old patient with acute unilateral optic neuritis are shown in Fig. (2). Her visual acuity was hand motion at 30 cm in the right and 20/15 in the left eyes. A relative afferent papillary defect was present in the right eye. The optic disc was slightly hyperemic and swollen in the right eye and was normal in the left eye. PVEP was extinguished when the pattern was presented to the right eye and normal in the left eye. The signal intensity ratios of optic nerve to frontal lobe are shown in Table 1. The signal intensity ratio of the MR images of the optic nerve of the affected right eye was higher than that of the left fellow eye at all of the 4 points behind the eye.

**Fig. (3):**
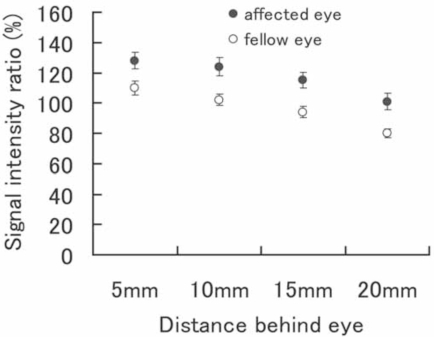
Means ± SE of the values from the 4 slices behind the eye are shown. In all parts, the ratio was significantly higher in affected eyes.

The mean and standard error of signal intensity ratio of the optic nerve relative to that of the frontal lobe at 4 points obtained from all of 25 patients is shown in Fig. (3). At all 4 points, the mean signal intensity ratio of the affected eyes is statistically significant higher than fellow eyes (p=0.003 at 5mm, p=0.001 at 10mm, p=0.0003 at 15mm and p=0.0002 at 20mm).

Sixteen of 25 patients with optic neuritis showed higher signal intensity at all 4 points in the affected eyes than the fellow eyes, and 23 of 25 patients showed higher mean signal intensity in the affected eye in the 4 portions. The 11 patients showed optic disc swelling in the affected eyes and in all these 11 eyes had a higher signal intensity at 5 mm behind the eye compared with the fellow eye.

When we define the normal range of the signal intensity ratio is within mean ± 2SD of that of fellow eyes, 12 patients had abnormally higher ratio at least one of 4 portions.

## DISCUSSION

Despite the high sensitivity of MRI in detecting brain lesions in patients with multiple sclerosis [8], the imaging and the detection of abnormalities of the optic nerve have proved to be unsatisfactory because of high signal intensity of orbital fat. There have been improvements in the MRI techniques to detect optic nerve lesions in patients with acute optic neuritis since Johnson *et al.* [1] introduced the excellent results with the MR STIR method. Evaluations were based on signal abnormalities, such as the signal intensity [3], the enlargement of the nerve width [11], the length and the position of the lesion [3, 6, 12-14]. The precise measurement of the size and length of optic nerve lesions is considered unreliable because of the oblique placement of the optic nerve in the coronal plane.

Such unreliability of the MR images of optic nerve lesions was partly overcome by STIR [1, 3], orbital fat suppression [2, 7], and the T2-weighted method [6]. Among these, the T2-weighted images can detect the edema of acute lesions and the gliosis and demyelination of chronic lesions. In patients with acute ON, signal intensity of optic nerve increases in T2-weighted MR images because of edema of optic nerve.

The evaluation of MRI abnormalities is usually facilitated by comparisons with the normal contralateral optic nerve or with the white matter of the brain. In general, the increased signal intensity of the optic nerve is judged without any quantitative calculations. For quantitative analysis, Davies *et al.* [8] reported quantitative ratios of the signal intensity of the optic nerve to the other tissues, orbital fat, frontal white matter and cerebrospinal fluid, and classified into 4 grades. In the present study, we measured signal intensity of optic nerve and white matter of the frontal lobe and calculated the ratio of signal intensity of optic nerve to that of white matter in the same plane for quantitative analysis, because of the histological similarity between optic nerve and white matter. We compared the ratio of the affected eyes to the fellow eyes. Though in case of ON in multiple sclerosis there may exist the hidden ON in the fellow eyes, we used the fellow eyes as control because there were no sign of optic neuritis even with PVEP. In order to increase the reliability of the method, the measurements were made at 4 parts of the retrobulbar portion of the optic nerve. We found a significantly higher signal intensity in the optic nerve of eyes diagnosed with ON than in the optic nerve of the normal fellow eyes in all 4 parts. And 12 of 25 patients (48%) showed statistically significantly higher signal intensity ratio at least one of 4 parts.

The Optic Neuritis Treatment Trial concluded that MR imaging has limited value in establishing the diagnosis of ON [15]. The authors recommended that MR imaging be performed for diagnostic purposes only when the clinical findings do not conform to the typical profile of optic neuritis. We agree with their conclusion because MR imaging is only one of the ways to detect abnormal lesions in the optic nerve.

## CONCLUSION

We reported a method to analyze optic nerve quantitatively by MR imaging and analyzed statistically compared to the fellow eyes in patients with unilateral optic neuritis. From our present results, we cannot refer to the sensitivity of our method, because we did not apply our present methods to other diseases. But with this method we think inter-image and inter-observer variability must reduce. And if MR images of same patients are obtained in the different stage of ON, this method must be useful. Further studies are required about the sensitivity and the relation between the optic nerve pathological condition and the signal intensity ratio.

## Figures and Tables

**Table 1.** Signal Intensity Ratio (%) in the Case Shown in Fig. (2)

	Distance from Eye			
	5mm	10mm	15mm	20mm
Right Eye	154.7	145.3	104.5	121.2
Left Eye	107.6	85.6	88.1	88.0
